# Supplementary material for: Comprehensive mutagenesis identifies the peptide repertoire of a p53 T-cell receptor mimic antibody that displays no toxicity in mice transgenic for human HLA-A*0201
Source: PLoS One. 2021 Apr 9;16(4):e0249967. doi: 10.1371/journal.pone.0249967 (PMC8034716; doi:10.1371/journal.pone.0249967)
Supplement: S1 File — (DOCX) [file pone.0249967.s001.docx]

**S1 File.**

**(Affinity of T1-116C Humanized Variants)**

**Quartz Crystal Microbalance (QCM)**

The affinity of humanized T1-116C antibody variants for their target p53RMP peptide/HLA*0201 complex was determined using a quartz crystal microbalance (QCM) assay performed by Lonza Biologics PLC. A polyclonal rabbit anti-human IgG antibody (Attana) was immobilized on an Attana LNB Carboxy Sensor Chip via amine coupling. The T1-116C humanized variants were captured and binding to the HLA-A2/p53 refolded protein was studied using an 8-point 1:2 serial dilution starting at a concentration of 50μg/ml. All measurements were performed at 25°C in 1xHBS-T running buffer. Data was collected by Attester software v3.0 and subsequently processed in Attester Evaluation v3.0 and Clamp XP software.

**(Specificity of T1-116C Humanised Variants)**

**T1-116C Humanization and Staining of Cell Lines**

T1-116C was humanized and deimmunized by Lonza Biologics PLC. *In silico* humanization and deimmunization were performed on heavy and light chain sequences using CDR grafting technology and T-cell epitope reduction. Heavy chain and light chain variable region cDNAs were synthesized and cloned into expression vectors encoding human IgG1 framework. Transient transfection was performed in CHOK1SV GS-KO cells and humanized antibodies were purified from 200ml culture supernatants via Protein A chromatography. T2 cells (pulsed with a wild type, alanine or glycine substituted p53RMP peptide) or cancer cell lines were washed with FACS wash, and incubated with primary antibody followed by secondary antibody as per the staining protocol described in the manuscript.
