# Supplementary material for: Comprehensive mutagenesis identifies the peptide repertoire of a p53 T-cell receptor mimic antibody that displays no toxicity in mice transgenic for human HLA-A*0201
Source: PLoS One. 2021 Apr 9;16(4):e0249967. doi: 10.1371/journal.pone.0249967 (PMC8034716; doi:10.1371/journal.pone.0249967)
Supplement: S1 Fig — (A) Binding of T1-116C humanized variants (V1-V4) to peptide-pulsed T2 cells and cancer cell lines including lymphoma OCI-Ly8, lung cancer NCI-H1395 and breast cancer MDA-MB-231. Flu peptide was used as a negative control. Blue is isotype control. Red is BB7.2 or one of the T1-116C antibodies. Peptides where (B) alanine or (C) glycine replaced the original amino acid at the indicated positions were used in T2 assays. MFI of each binding was normalized against T1-116C binding to the original p53RMP peptide. In (C) amino acids at positions 5 and 6 of p53RMP are composed of alanine therefore no alanine replacement was performed at these positions. Humanization retained binding specificity to the HLA-A2-p53RMP complex at the protein level. The humanized T1‐116CV1 variant was selected for future experiments because it performs most comparably to the original murine T1‐116C. (PPTX) [file pone.0249967.s002.pptx]

## Slide 1
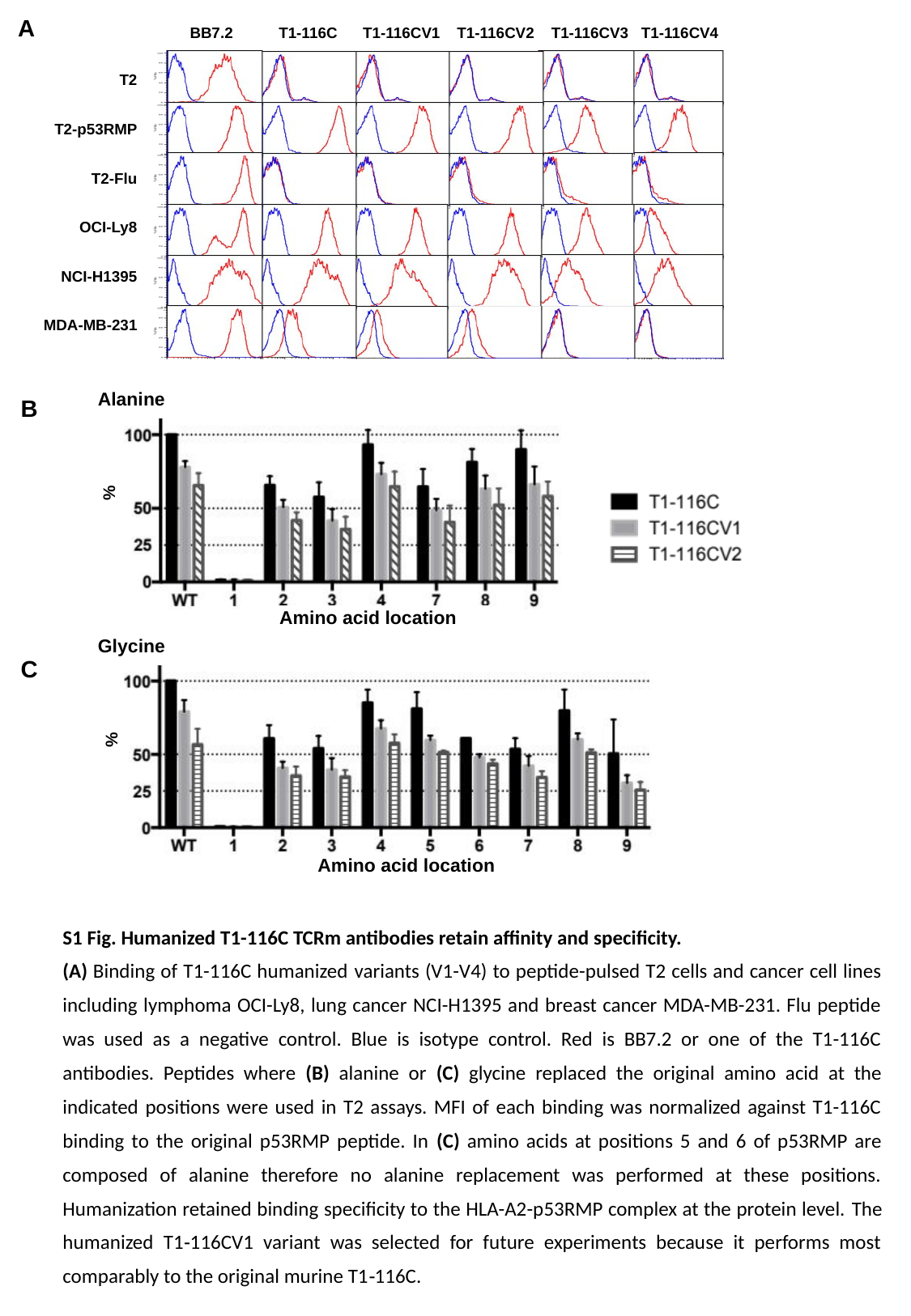

A
 BB7.2	 T1-116C T1-116CV1 T1-116CV2 T1-116CV3 T1-116CV4
T2
T2-p53RMP
T2-Flu
OCI-Ly8
NCI-H1395
MDA-MB-231
Alanine
%
Amino acid location
B
Glycine
%
Amino acid location
C
S1 Fig. Humanized T1-116C TCRm antibodies retain affinity and specificity.
(A) Binding of T1-116C humanized variants (V1-V4) to peptide-pulsed T2 cells and cancer cell lines including lymphoma OCI-Ly8, lung cancer NCI-H1395 and breast cancer MDA-MB-231. Flu peptide was used as a negative control. Blue is isotype control. Red is BB7.2 or one of the T1-116C antibodies. Peptides where (B) alanine or (C) glycine replaced the original amino acid at the indicated positions were used in T2 assays. MFI of each binding was normalized against T1-116C binding to the original p53RMP peptide. In (C) amino acids at positions 5 and 6 of p53RMP are composed of alanine therefore no alanine replacement was performed at these positions. Humanization retained binding specificity to the HLA-A2-p53RMP complex at the protein level. The humanized T1‐116CV1 variant was selected for future experiments because it performs most comparably to the original murine T1‐116C.
