# Supplementary material for: Comprehensive mutagenesis identifies the peptide repertoire of a p53 T-cell receptor mimic antibody that displays no toxicity in mice transgenic for human HLA-A*0201
Source: PLoS One. 2021 Apr 9;16(4):e0249967. doi: 10.1371/journal.pone.0249967 (PMC8034716; doi:10.1371/journal.pone.0249967)
Supplement: S1 Table — (PPTX) [file pone.0249967.s003.pptx]

## Slide 1
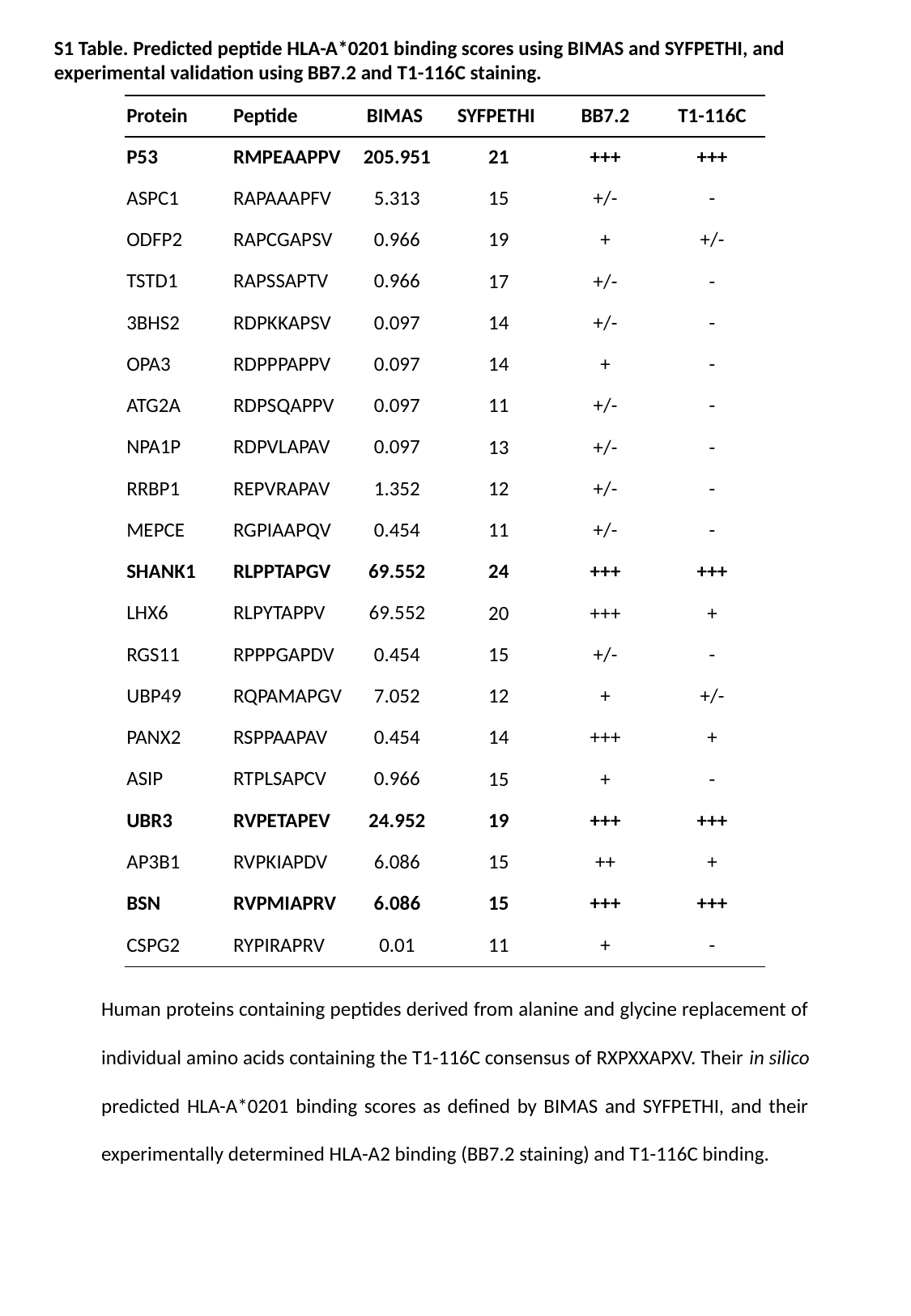

S1 Table. Predicted peptide HLA-A*0201 binding scores using BIMAS and SYFPETHI, and experimental validation using BB7.2 and T1-116C staining.
| Protein | Peptide | BIMAS | SYFPETHI | BB7.2 | T1-116C |
| --- | --- | --- | --- | --- | --- |
| P53 | RMPEAAPPV | 205.951 | 21 | +++ | +++ |
| ASPC1 | RAPAAAPFV | 5.313 | 15 | +/- | - |
| ODFP2 | RAPCGAPSV | 0.966 | 19 | + | +/- |
| TSTD1 | RAPSSAPTV | 0.966 | 17 | +/- | - |
| 3BHS2 | RDPKKAPSV | 0.097 | 14 | +/- | - |
| OPA3 | RDPPPAPPV | 0.097 | 14 | + | - |
| ATG2A | RDPSQAPPV | 0.097 | 11 | +/- | - |
| NPA1P | RDPVLAPAV | 0.097 | 13 | +/- | - |
| RRBP1 | REPVRAPAV | 1.352 | 12 | +/- | - |
| MEPCE | RGPIAAPQV | 0.454 | 11 | +/- | - |
| SHANK1 | RLPPTAPGV | 69.552 | 24 | +++ | +++ |
| LHX6 | RLPYTAPPV | 69.552 | 20 | +++ | + |
| RGS11 | RPPPGAPDV | 0.454 | 15 | +/- | - |
| UBP49 | RQPAMAPGV | 7.052 | 12 | + | +/- |
| PANX2 | RSPPAAPAV | 0.454 | 14 | +++ | + |
| ASIP | RTPLSAPCV | 0.966 | 15 | + | - |
| UBR3 | RVPETAPEV | 24.952 | 19 | +++ | +++ |
| AP3B1 | RVPKIAPDV | 6.086 | 15 | ++ | + |
| BSN | RVPMIAPRV | 6.086 | 15 | +++ | +++ |
| CSPG2 | RYPIRAPRV | 0.01 | 11 | + | - |
Human proteins containing peptides derived from alanine and glycine replacement of individual amino acids containing the T1-116C consensus of RXPXXAPXV. Their in silico predicted HLA-A*0201 binding scores as defined by BIMAS and SYFPETHI, and their experimentally determined HLA-A2 binding (BB7.2 staining) and T1-116C binding.
