# Supplementary material for: Comprehensive mutagenesis identifies the peptide repertoire of a p53 T-cell receptor mimic antibody that displays no toxicity in mice transgenic for human HLA-A*0201
Source: PLoS One. 2021 Apr 9;16(4):e0249967. doi: 10.1371/journal.pone.0249967 (PMC8034716; doi:10.1371/journal.pone.0249967)
Supplement: S3 Table — (PPTX) [file pone.0249967.s005.pptx]

## Slide 1
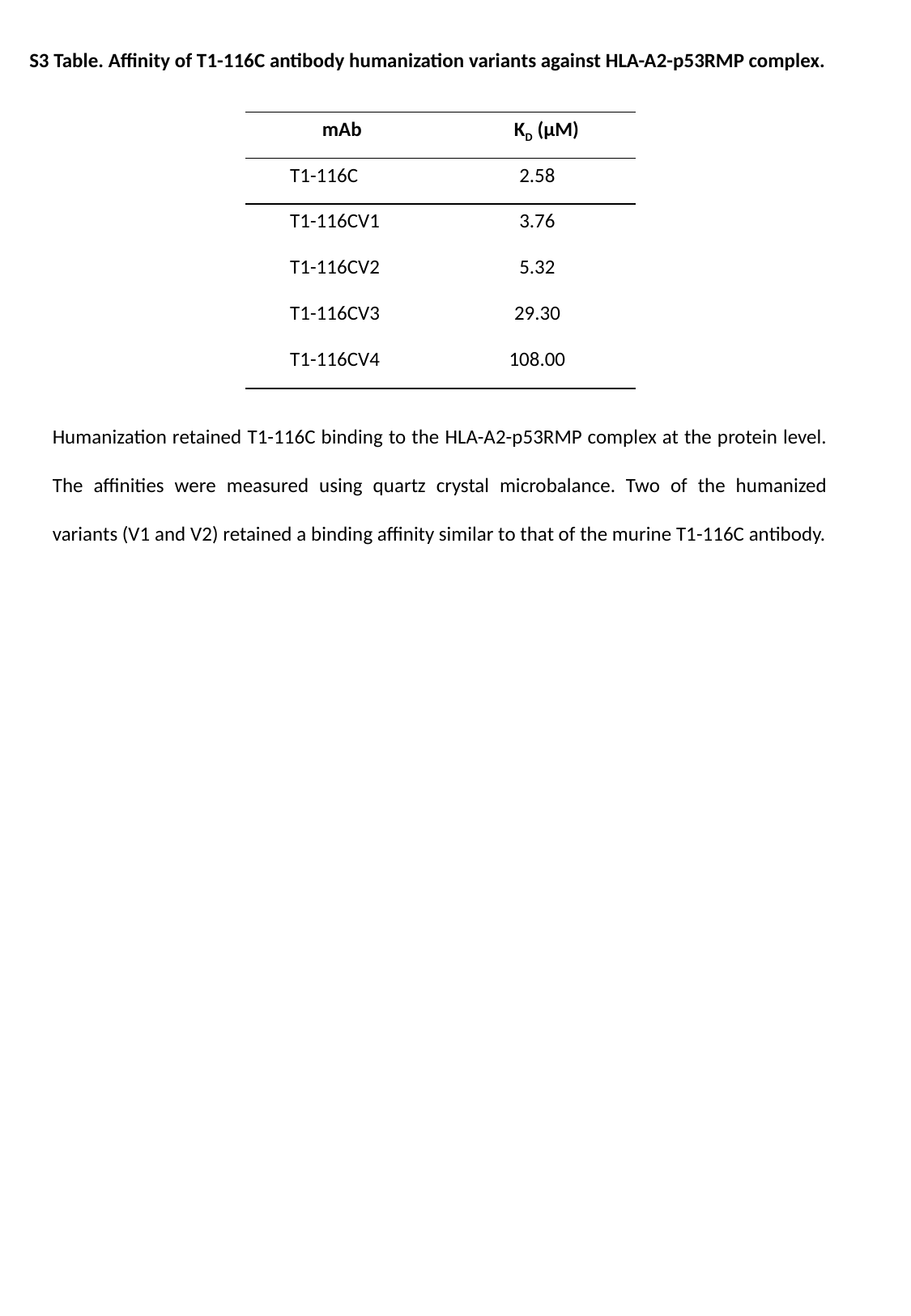

S3 Table. Affinity of T1-116C antibody humanization variants against HLA-A2-p53RMP complex.
| mAb | KD (μM) |
| --- | --- |
| T1-116C | 2.58 |
| T1-116CV1 | 3.76 |
| T1-116CV2 | 5.32 |
| T1-116CV3 | 29.30 |
| T1-116CV4 | 108.00 |
Humanization retained T1-116C binding to the HLA-A2-p53RMP complex at the protein level. The affinities were measured using quartz crystal microbalance. Two of the humanized variants (V1 and V2) retained a binding affinity similar to that of the murine T1-116C antibody.
