# Supplementary material for: Comprehensive mutagenesis identifies the peptide repertoire of a p53 T-cell receptor mimic antibody that displays no toxicity in mice transgenic for human HLA-A*0201
Source: PLoS One. 2021 Apr 9;16(4):e0249967. doi: 10.1371/journal.pone.0249967 (PMC8034716; doi:10.1371/journal.pone.0249967)
Supplement: S4 Table — (PPTX) [file pone.0249967.s006.pptx]

## Slide 1
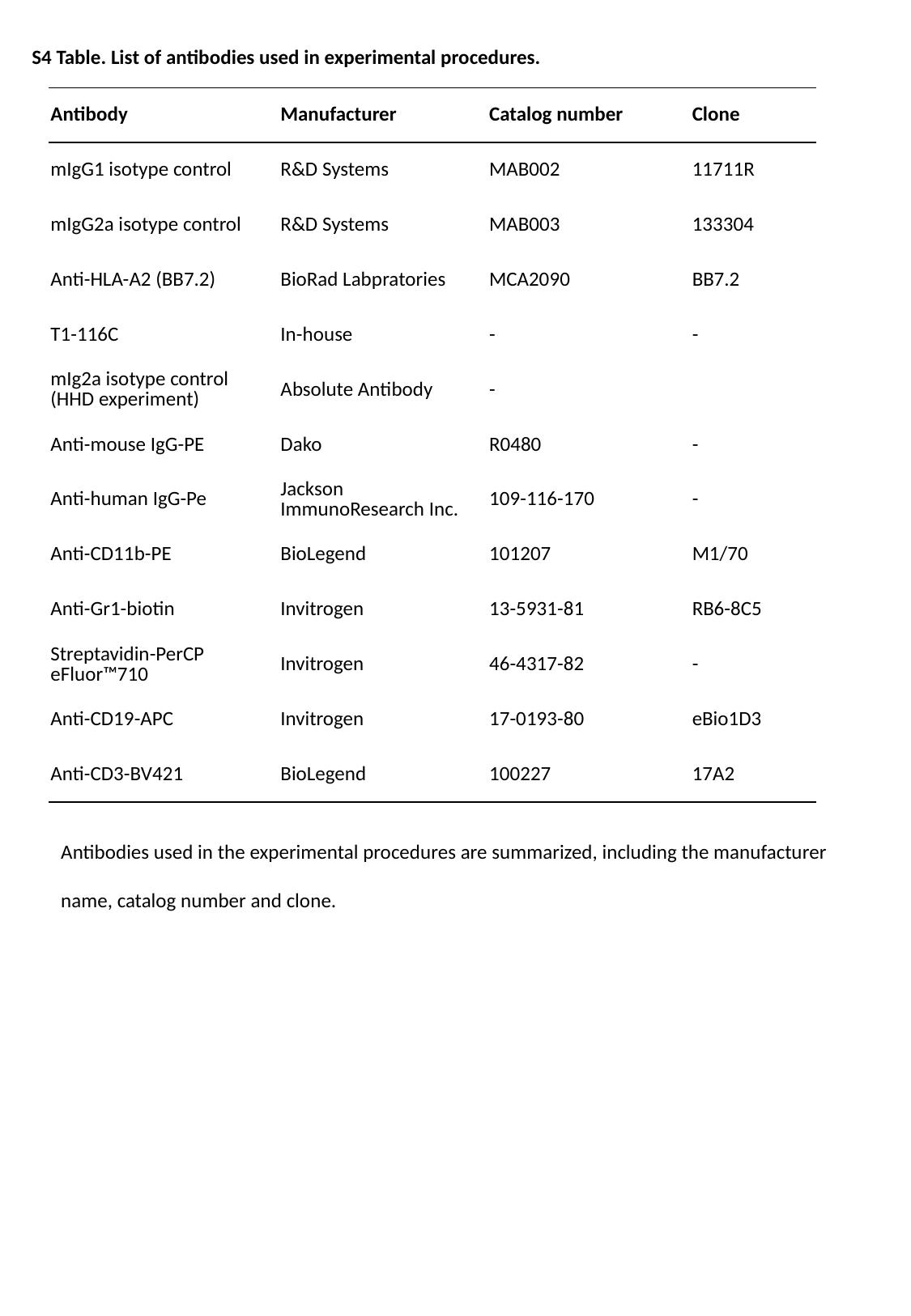

S4 Table. List of antibodies used in experimental procedures.
| Antibody | Manufacturer | Catalog number | Clone |
| --- | --- | --- | --- |
| mIgG1 isotype control | R&D Systems | MAB002 | 11711R |
| mIgG2a isotype control | R&D Systems | MAB003 | 133304 |
| Anti-HLA-A2 (BB7.2) | BioRad Labpratories | MCA2090 | BB7.2 |
| T1-116C | In-house | - | - |
| mIg2a isotype control (HHD experiment) | Absolute Antibody | - | |
| Anti-mouse IgG-PE | Dako | R0480 | - |
| Anti-human IgG-Pe | Jackson ImmunoResearch Inc. | 109-116-170 | - |
| Anti-CD11b-PE | BioLegend | 101207 | M1/70 |
| Anti-Gr1-biotin | Invitrogen | 13-5931-81 | RB6-8C5 |
| Streptavidin-PerCP eFluor™710 | Invitrogen | 46-4317-82 | - |
| Anti-CD19-APC | Invitrogen | 17-0193-80 | eBio1D3 |
| Anti-CD3-BV421 | BioLegend | 100227 | 17A2 |
Antibodies used in the experimental procedures are summarized, including the manufacturer name, catalog number and clone.
