# Supplementary material for: Comprehensive mutagenesis identifies the peptide repertoire of a p53 T-cell receptor mimic antibody that displays no toxicity in mice transgenic for human HLA-A*0201
Source: PLoS One. 2021 Apr 9;16(4):e0249967. doi: 10.1371/journal.pone.0249967 (PMC8034716; doi:10.1371/journal.pone.0249967)
Supplement: S5 Table — (PPTX) [file pone.0249967.s007.pptx]

## Slide 1
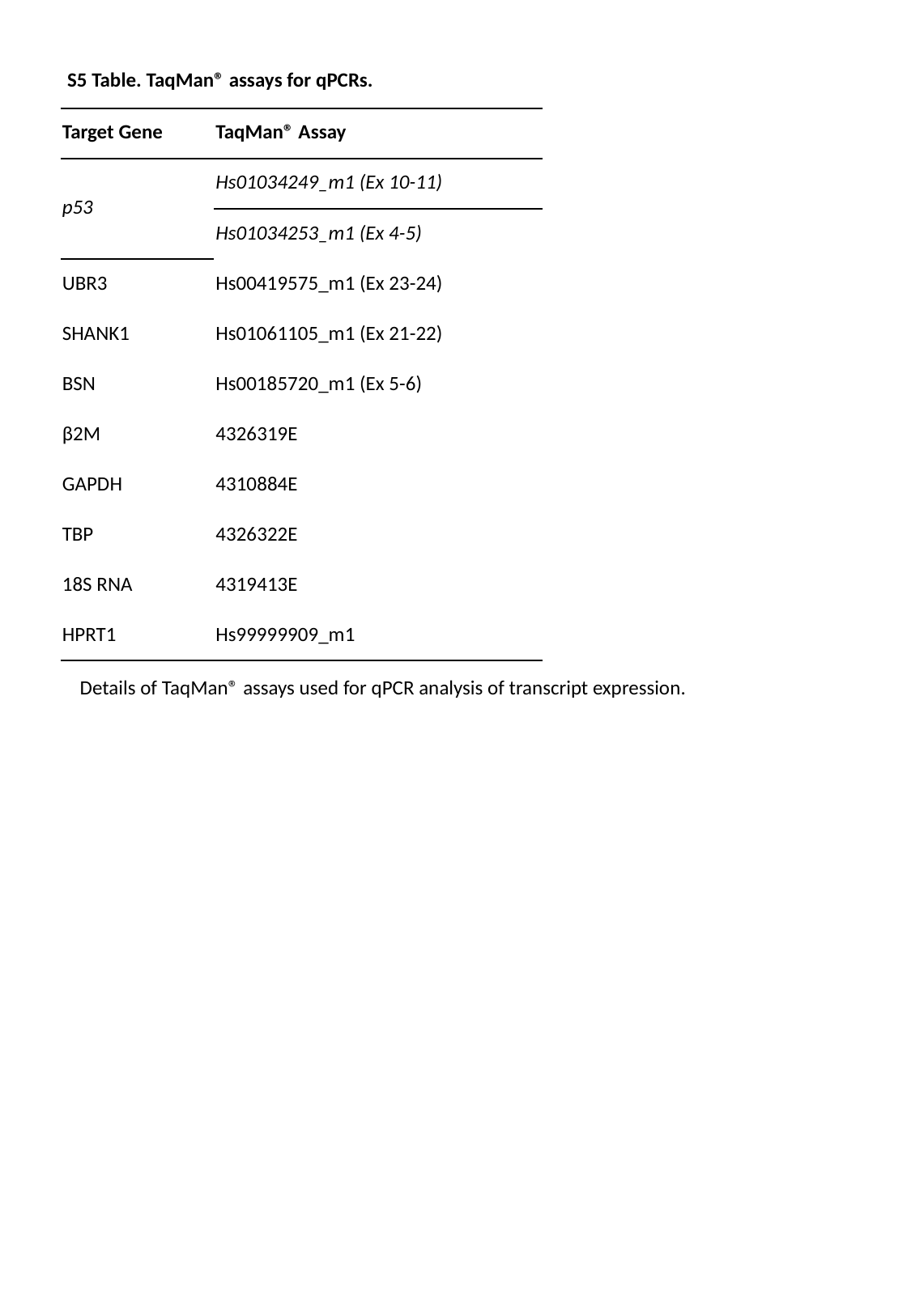

S5 Table. TaqMan® assays for qPCRs.
| Target Gene | TaqMan® Assay |
| --- | --- |
| p53 | Hs01034249\_m1 (Ex 10-11) |
| | Hs01034253\_m1 (Ex 4-5) |
| UBR3 | Hs00419575\_m1 (Ex 23-24) |
| SHANK1 | Hs01061105\_m1 (Ex 21-22) |
| BSN | Hs00185720\_m1 (Ex 5-6) |
| β2M | 4326319E |
| GAPDH | 4310884E |
| TBP | 4326322E |
| 18S RNA | 4319413E |
| HPRT1 | Hs99999909\_m1 |
Details of TaqMan® assays used for qPCR analysis of transcript expression.
